# Predicting Gleason Score of Prostate Cancer Patients Using Radiomic Analysis

**DOI:** 10.3389/fonc.2018.00630

**Published:** 2018-12-18

**Authors:** Ahmad Chaddad, Tamim Niazi, Stephan Probst, Franck Bladou, Maurice Anidjar, Boris Bahoric

**Affiliations:** ^1^Division of Radiation Oncology, McGill University, Montreal, QC, Canada; ^2^Department of Automated Production Engineering, ETS, Montreal, QC, Canada; ^3^Division of Nuclear Medicine, McGill University, Montreal, QC, Canada; ^4^Depatment of Urology, McGill University, Montreal, QC, Canada

**Keywords:** biomarkers, classification, gleason score, radiomics, prostate cancer

## Abstract

**Purpose:** Use of quantitative imaging features and encoding the intra-tumoral heterogeneity from multi-parametric magnetic resonance imaging (mpMRI) for the prediction of Gleason score is gaining attention as a non-invasive biomarker for prostate cancer (PCa). This study tested the hypothesis that radiomic features, extracted from mpMRI, could predict the Gleason score pattern of patients with PCa.

**Methods:** This analysis included T2-weighted (T2-WI) and apparent diffusion coefficient (ADC, computed from diffusion-weighted imaging) scans of 99 PCa patients from The Cancer Imaging Archive (TCIA). A total of 41 radiomic features were calculated from a local tumor sub-volume (i.e., regions of interest) that is determined by a centroid coordinate of PCa volume, grouped based on their Gleason score patterns. Kruskal-Wallis and Spearman's rank correlation tests were used to identify features related to Gleason score groups. Random forest (RF) classifier model was used to predict Gleason score groups and identify the most important signature among the 41 radiomic features.

**Results:** Gleason score groups could be discriminated based on zone size percentage, large zone size emphasis and zone size non-uniformity values (*p* < 0.05). These features also showed a significant correlation between radiomic features and Gleason score groups with a correlation value of −0.35, 0.32, 0.42 for the large zone size emphasis, zone size non-uniformity and zone size percentage, respectively (corrected *p* < 0.05). RF classifier model achieved an average of the area under the curves of the receiver operating characteristic (ROC) of 83.40, 72.71, and 77.35% to predict Gleason score groups (G1) = 6; 6 < (G2) < (3 + 4) and (G3) ≥ 4 + 3, respectively.

**Conclusion:** Our results suggest that the radiomic features can be used as a non-invasive biomarker to predict the Gleason score of the PCa patients.

## Introduction

Prostate cancer (PCa) is one of the most prevalent male malignancies in the developed countries and 1/6th of the men in the USA are expected to be diagnosed with this disease in their lifetime (1). Patients with localized PCa are classified into three risk groups (low, intermediate, and high risk) based on their prostate-specific Antigen (PSA) level, Gleason score and clinical stage (i.e., TNM) (2). For men with low-risk prostate cancer, active surveillance as opposed to immediate treatment has become a widely accepted treatment approach (3, 4). However, PCa has the capacity to progress over time. A study of 17,943 patients with low-risk PCa who were treated with radical prostatectomy (RP) revealed that upgrading and upstaging occurred in 45% of these men (5). Furthermore, the deferral of RP for more than 12 months has been associated with a 1.7-fold increased risk of non-organ confined disease after surgery (5).

Numerous studies have shown that PSA alone is not an accurate indicator of PCa and over-diagnosis is occurring in up to 50% of men (6, 7). The inaccuracy of PSA screening often leads to subsequent unnecessary biopsies that are not only costly but invasive with serious side effects, such as infection, erectile dysfunction and bleeding. Therefore, there is a pressing need for a non-invasive test to distinguish between PCa grades to improve delivery of high precision care for these patients.

MR imagery, especially multi-parametric sequences (i.e., T1-WI, T2-WI, FLAIR … etc.), have been widely used for diagnosis, staging and treatment monitoring of different tumor types (8–15). A newly structured Prostate Imaging Reporting and Data System (PI-RADS v2) showed that this scoring method could predict the risk of prostate cancer presence based on the MR images (16). However, PI-RADS v2 involves interpretation of the images based on the experience of the radiologists that carries inter-reader variability. Moreover, PI-RADS has been used to investigate the relation between imaging features and Gleason score (GS) (9, 10, 17, 18). The volume of PCa has been shown to be significantly different between GS = 6 and GS ≥ 7 PCa (17). In addition, two recent studies demonstrated the usefulness of the texture features based on gray level co-occurrence matrices (GLCMs) of MR images (i.e., T2WI) as an indicator for the pathological differences in PCa (19, 20). To date, there has been limited work investigating the link between the texture features of the PCa imaging and the GS.

Offering a non-invasive and low-cost automated technique for analysis of tumor properties based on MR images, radiomics has recently been used to interrogate the tumor heterogeneity of several types of cancer, such as GBM (21), lung (22, 23), colorectal (24, 25), and PCa (17, 26–28) among others. Radiomic features with machine learning models can analyze large numbers of PCa images that will overcome the limitations (i.e., inter-reader variability for interpretation of the ROIs within an image) for assessing and classifying PCa lesions. Specifically, many previous studies have used such machine learning models to assess for the PCa aggressiveness (13, 29–32). Combined structural features and metabolic imaging data of PCa in mpMRI [i.e., T2-WI and magnetic resonance spectroscopy (MRS)] using the RF classifier, have shown the capacity of these models to detect the areas of cancer in a PCa tissue (18, 33). Extracted multi-texture features derived from T1-WI, T2-WI, and Diffusion-Weighted Imaging (DWI) to characterize the PCa tissue have been demonstrated to improve the classification rate of prostate tumor recognition (34–36). Differences between the cancer grades of a tumor (i.e., PCa) vs. non-tumor regions have been related to differences in heterogeneity (i.e., texture). For example, PCa can be discriminated from benign tissues and be detected based on a different histogram analysis, as shown by Vos et al (37). However, the dominant features that could assist in measuring the heterogeneity within PCa have not yet been fully studied.

We hypothesize that the comprehensive integration of radiomic features from mpMR images will identify new characteristics that are capable of distinguishing PCa with different GS groups whether in the transition or peripheral zones.

## Materials and Methods

**Figure 1:**
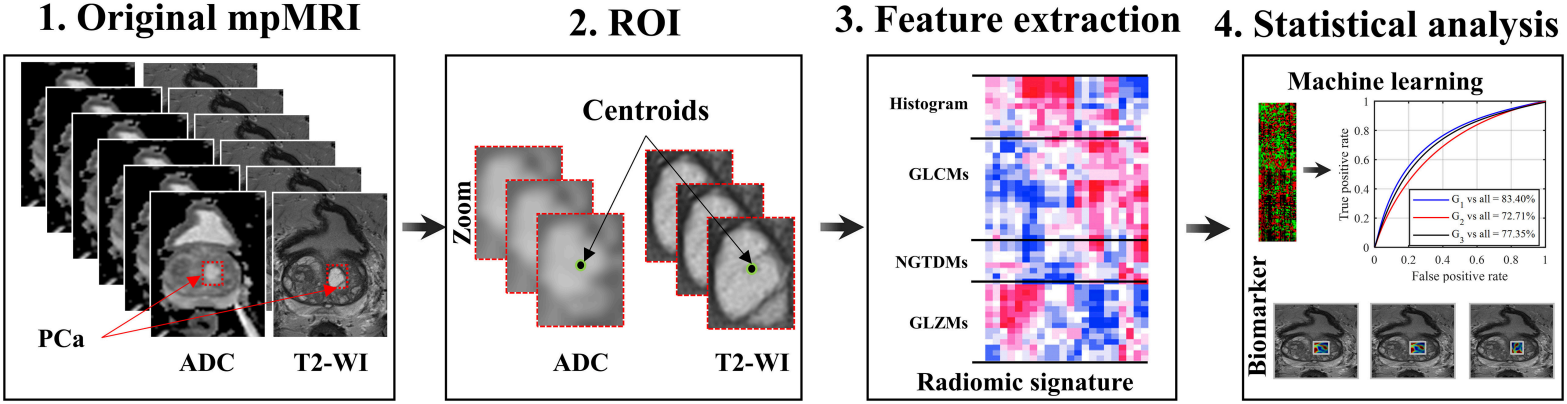
Schema of a radiomic model for patients with PCa. Acquisition of pre-treatment PCa patient's MR images; Regions of interest (i.e., subvolume 21 × 21 × 3 voxels); Extraction of 41 radiomic features from ROIs; Feature significance analysis based on Spearman rank correlation and Kruskal-Wallis, and multivariate prediction of Gleason score groups using the random forest model.

The proposed pipeline of radiomic analysis to predict GS involves data acquisition from T2-WI and ADC images, automatic matching of PCa regions, radiomic features computation from determined subvolume of PCa tumor, and feature analysis. The proposed flowchart for radiomic analysis of PCa is shown in Figure 1. Kruskal-Wallis significance test and Spearman rank correlation were performed to identify radiomic features which were associated with GS groups. We then applied the RF classifier, using the radiomic features, to differentiate between the GS groups and rank the importance value of each radiomic features for prediction. The detailed methodology for each step of the proposed flowchart is described below.

### Patients and Data Acquisition

We reviewed the 99 PCa patients of the SPIE-AAPM-NCI Prostate MR Gleason Grade Group Challenge (http://spiechallenges.cloudapp.net/competitions/7) and the Cancer Imaging Archive (TCIA), a publicly available medical image repository. Note that the challenge consists of 162 PCa (99 training and 63 testing) patients. We considered only the 99 training cases where the Gleason score was available, previously de-identified by SPIE-AAPM-NCI, and the dataset is available for public download (i.e., Supplementary Table 1). The remaining 63 cases (i.e., testing) did not have Gleason score available. As such, no institutional review board or Health Insurance Portability and Accountability Act approval were required for our study. The dataset included T2-WI and ADC which were computed from DWI. The MR images were acquired on two different types of Siemens 3T MR scanners, the MAGNETOM Trio and Skyra. T2-weighted images were acquired using a turbo spin echo sequence and had a resolution of around 0.5 mm in the plane and a slice thickness of 3.6 mm. The DWI series were acquired with a single-shot echo planar imaging sequence with a resolution of 2 mm in-plane and 3.6 mm slice thickness with diffusion-encoding gradients in three directions. Three b-values were acquired (50, 400, and 800), and the ADC map was subsequently calculated by the scanner software (https://wiki.cancerimagingarchive.net/). Gray-scale images were then intensity normalized to reduce the intensity variation between MRIs obtained from different acquisitions. Moreover, images were acquired at a resolution of 320 × 320 × 19 voxels. Patient characteristics information is reported in Supplementary Table 2. The histograms of voxel intensity distribution across the T2-WI and ADC are not reliable for differentiating between the three Gleason Groups, (i.e., Supplementary Figure 1). The five groups of Gleason scores were divided into three groups—G1 when GS ≤ 6; G2 when GS = 3 + 4; and G3 when GS ≥ 4 + 3. Therefore, we used several texture features derived from gray-level co-occurrence matrix (GLCM), neighborhood gray-tone difference matrix (NGTDM) and gray-level size zone matrix (GLSZM) that able to capture the subtle differences between the GS groups.

### Feature Extraction

Regions of interest (ROI) of PCa were automatically selected based on the centroid coordinates of all the lesions in T2-WI and ADC images that were provided by the SPIE-AAPM-NCI Prostate MR Gleason Grade Group Challenge. For each patient, a sub-volume (i.e., ROI) of 21 × 21 × 3 voxels was collected separately, from the axial image in T2-WI and ADC to ensure accuracy and precision. Each sub-volume is encoded into a set of features by applying the 41 radiomic features as following: six intensity features derived from histogram and 35 texture features derived from GLCMs (38, 39), NGTDM, (40), GLSZM (41) as shown in Supplementary Table 3.

These features measure various textural properties and quantify the hidden patterns in the ROI. To capture more meaningful patterns of texture, image intensities of ROIs were uniformly quantized to 32 gray levels prior to computing the features. These features are described in several previous studies (22, 38–41) and detailed description of each feature is listed in their Supplementary Materials (22, 42). Features were extracted separately from T2-WI and ADC images and the average of each feature across the two images (i.e., T2-WI and ADC) was considered.

### Statistical Analysis

To identify the significant radiomic features in comparing between the three groups of GS (i.e., G1, G2, G3), we first applied the Kruskal-Wallis test on each radiomic feature before multiple corrections using the Holm-Bonferroni method (43) to identify significant features (*P* < 0.05). We then used Spearman's rank correlation (44) to compute the correlation value (ρ) between each of radiomic features and the GS group for the corresponding PCa patients. The rank correlation value was obtained between the GS group and each radiomic feature, with values between ±0.3 and ±0.5 indicating moderate (i.e., middle) correlation. We measured the significance of these correlation values based on the null hypothesis that there is no correlation. As in the previous test, we corrected *p*-values using the Holm-Bonferroni procedure and considered correlation significant if they reached a *p* < 0.05 after correction.

We considered all 41 radiomic features as the input for the RF classifier model (45) and performed multivariate analysis for classifying the patients into three groups of GS [G1 vs. all (G2-G3); G2 vs. all (G1-G3); G3 vs. all (G1-G2)]. We utilized RF for our analysis since it is one of the most effective classification models and leads to a low bias/variance classification result. In addition, the RF model in training algorithm possesses a feature selection process that allows assessment of each input feature (46). We acknowledge however that various other classifier models could be used for this task.

To report unbiased metrics, we used a 5-fold cross-validation strategy, where training features are divided into 5 equal sized subsets and, in each validation run, one subset is put aside for testing and the remaining 4 subsets are used to train the RF classifier. Each of these subsets was used in turn to compute the performance metrics of the trained RF model of the remaining samples using the 500 decision trees [i.e., number of trees within the RF model (47)]. Performance metrics [i.e., area under the curve (AUC), classifier accuracy, negative predictive value (NPV) and positive predictive value (PPV)], are then reported as the average obtained across 5-folds.

To compute the importance of each of the 41 features in individual classification group [G1 vs. all (G2-G3); G2 vs. all (G1-G3); G3 vs. all (G1-G2)], we measured the increase in prediction error resulting from the permutation of feature values across out-of-bag observations. The importance values were computed for every RF tree and averaged over the entire ensemble. These values were then normalized by dividing them by the ensemble's standard deviation. Finally, the importance of each of the 41 features was obtained by averaging these normalized values across all 5-folds. Positive importance value indicates that the feature is predictive, whereas negative importance value identifies features with no predictive value.

## Results

### Patients Characteristics and Data Acquisition

In this study we used a retrospective dataset comprising of 99 PCa patients collected from the SPIE-AAPM-NCI and the Cancer Imaging Archive (TCIA). Each patient's tumor lesion has a pathology Gleason Grade Group (GGG) number, which consists of five groups defined previously by Esptein JI et al. (48). However, we reclassified the patients based on their GGG into three groups to better represent clinical management: Group 1 (G1), 30 patients, Gleason score 6; Group 2 (G2), 39 patients, Gleason score 3 + 4; and Group 3 (G3), 30 patients, Gleason primary pattern of 4 or higher (4 + 3, 8, 9 or 10). The relevant patient characteristics are reported in Supplementary Table 2.

### Radiomic Features and Association With Gleason Score

After extracting 41 radiomic features from MR images of each PCa patient, we applied univariate analysis using the Kruskal-Wallis significance test to determine if any individual radiomic feature was statistically significant to compare between the GS groups. We also computed the Spearman's rank correlation coefficient between the radiomic features and GS groups.

**Figure 2:**
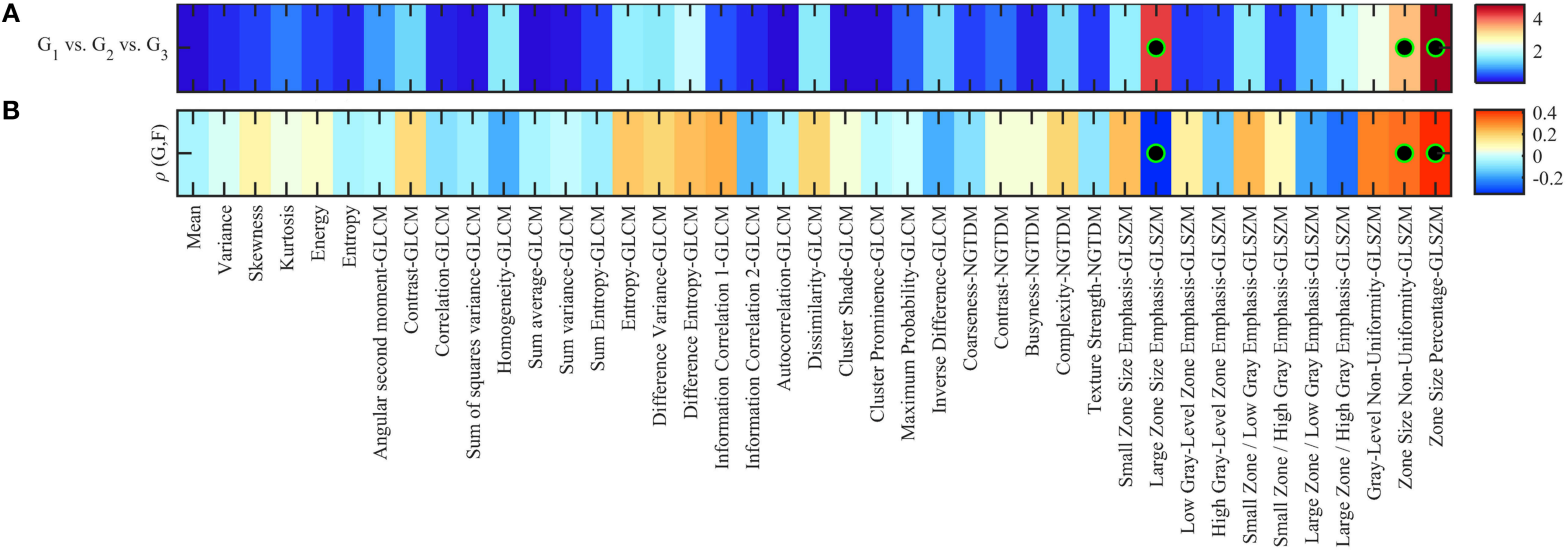
**(A)** Heatmap of Kruskal-Wallis significance test *p*-values (–log_10_ scale) using radiomic features to identify patients of different Gleason scores. Significant features to compare between Gleason score groups are indicated with a black-green circle (corrected *p* < 0.05). **(B)** Spearman rank correlation between feature value and groups of Gleason score (i.e., 1, 2, 3), color-coded from minimum (dark blue) to maximum (dark red). Features with statistically significant correlation (i.e., corrected *p* < 0.05) are indicated with a black-green circle.

In Kruskal-Wallis significance test, we found only three features, namely, large zone size emphasis, zone size non-uniformity and zone size percentage, that were statistically significant difference between the three GS groups (G1, G2 and G3): *p* < 0.05. None of the remaining features were statistically significant to compare between GS groups following Holm-Bonferroni correction (Figure 2A; Table 1). Spearman's rank correlation, applied between radiomic features and GS groups showed the significant moderate correlation values (ρ) of −0.35, 0.32, and 0.42 for the large zone size emphasis, zone size non-uniformity and zone size percentage, respectively with a corrected *p* < 0.05. The correlation values of remaining radiomic features were not statistically significant following Holm-Bonferroni correction (Figure 2B; Table 2). The significantly correlated features were similar to those which could distinguish between GS groups.

**Table 1:** Comparisons of radiomic features related to the Gleason score groups of prostate cancer.

**Radiomic features**	**Median (IQR: interquartile range)**			***P***
	**G1**	**G2**	**G3**
	***N* = 30**	***N* = 39**	***N* = 30**
**HISTOGRAM**				
Mean	0.410 (0.303)	0.494 (0.344)	0.448 (0.180)	0.88
Variance	0.481 (0.217)	0.434 (0.280)	0.357 (0.200)	0.53
Skewness	0.376 (0.285)	0.368 (0.218)	0.369 (0.208)	0.36
Kurtosis	0.068 (0.064)	0.064 (0.058)	0.065 (0.076)	0.17
Energy	0.365 (0.327)	0.313 (0.289)	0.291 (0.317)	0.38
Entropy	0.552 (0.258)	0.658 (0.252)	0.602 (0.298)	0.47
**GRAY LEVEL CO-OCCURRENCE MATRIX (GLCM)**				
Angular second moment	0.314 (0.268)	0.264 (0.225)	0.224 (0.236)	0.13
Contrast	0.285 (0.254)	0.388 (0.307)	0.390 (0.330)	0.04
Correlation	0.573 (0.255)	0.490 (0.376)	0.438 (0.407)	0.63
Sum of squares variance	0.408 (0.356)	0.410 (0.353)	0.399 (0.197)	0.79
Homogeneity	0.517 (0.139)	0.434 (0.289)	0.392 (0.160)	0.03
Sum average	0.402 (0.342)	0.440 (0.325)	0.438 (0.212)	0.87
Sum variance	0.477 (0.255)	0.426 (0.312)	0.342 (0.233)	0.68
Sum Entropy	0.585 (0.261)	0.706 (0.253)	0.649 (0.295)	0.39
Entropy	0.575 (0.283)	0.702 (0.298)	0.698 (0.295)	0.01
Difference variance	0.299 (0.303)	0.425 (0.357)	0.402 (0.471)	0.03
Difference entropy	0.469 (0.191)	0.576 (0.344)	0.596 (0.260)	0.01
Information correlation 1	0.567 (0.147)	0.623 (0.313)	0.625 (0.254)	0.03
Information correlation 2	0.619 (0.264)	0.577 (0.377)	0.496 (0.375)	0.22
Autocorrelation	0.414 (0.353)	0.413 (0.337)	0.414 (0.192)	0.82
Dissimilarity	0.404 (0.177)	0.502 (0.294)	0.526 (0.241)	0.03
Cluster shade	0.539 (0.180)	0.501 (0.163)	0.494 (0.220)	0.87
Cluster prominence	0.489 (0.440)	0.417 (0.334)	0.332 (0.346)	0.95
Maximum probability	0.360 (0.283)	0.312 (0.198)	0.306 (0.323)	0.27
Inverse difference	0.492 (0.152)	0.395 (0.283)	0.372 (0.153)	0.03
**NEIGHBORHOOD GRAY-TONE DIFFERENCE MATRIX (NGTDM)**				
Coarseness	0.012 (0.011)	0.009 (0.009)	0.010 (0.010)	0.37
Contrast	0.272 (0.159)	0.321 (0.258)	0.254 (0.123)	0.20
Busyness	0.359 (0.287)	0.374 (0.148)	0.384 (0.172)	0.65
Complexity	0.330 (0.232)	0.412 (0.199)	0.356 (0.258)	0.04
Texture Strength	0.124 (0.115)	0.088 (0.068)	0.097 (0.092)	0.46
**GRAY-LEVEL ZONE SIZE MATRIX (GLZSM)**				
Small zone size emphasis	0.515 (0.249)	0.585 (0.313)	0.615 (0.275)	0.02
Large zone size emphasis	0.339 (0.182)	0.194 (0.166)	0.226 (0.152)	*6.1 × 10^−5^
Low gray-level zone emphasis	0.231 (0.234)	0.273 (0.219)	0.306 (0.305)	0.41
High gray-level zone emphasis	0.583 (0.305)	0.539 (0.150)	0.499 (0.223)	0.36
Small zone/low gray emphasis	0.222 (0.190)	0.292 (0.246)	0.298 (0.191)	0.03
Small zone/high gray emphasis	0.361 (0.424)	0.403 (0.365)	0.400 (0.214)	0.41
Large zone/low gray emphasis	0.234 (0.181)	0.164 (0.143)	0.154 (0.163)	0.08
Large zone/high gray emphasis	0.114 (0.153)	0.076 (0.061)	0.084 (0.098)	0.01
Gray-level non-uniformity	0.381 (0.178)	0.396 (0.246)	0.487 (0.250)	4 × 10^−3^
Zone size non-uniformity	0.311 (0.158)	0.292 (0.179)	0.361 (0.200)	*3.8 × 10^−4^
Zone size percentage	0.361 (0.107)	0.369 (0.155)	0.404 (0.257)	*1.3 × 10^−5^

*Significant features following Holm-Bonferroni correction; G1, G2, and G3 is the Gleason score of the group 1, 2, and 3, respectively.

**Table 2:** Correlated features with Gleason score groups.

**Features**	**ρ (*p*)**
Large zone size emphasis-GLSZM	−0.35 (3.1 × 10^−4*^)
Zone size non-uniformity	0.32 (9.01 × 10^−4*^)
Zone size percentage-GLSZM	0.42 (1.1 × 10^−5*^)

* *Significant features following Holm-Bonferroni correction; ρ is the correlation coefficient*.

In order to show the changes in the original five Gleason score groups, we repeated the Kruskal-Wallis significance test and the Spearman correlation. Results can be found in Supplementary Figure 2, which shows the *p*-values in log_10_ scale and the correlation value. In general, two radiomic features were statistically significant to be associated with five GS groups [large zone size emphasis (ρ = −0.35) and zone size percentage (ρ = 0.43)] which are similar to those obtained using the three GS groups.

### Classification of GS Groups

**Figure 3:**
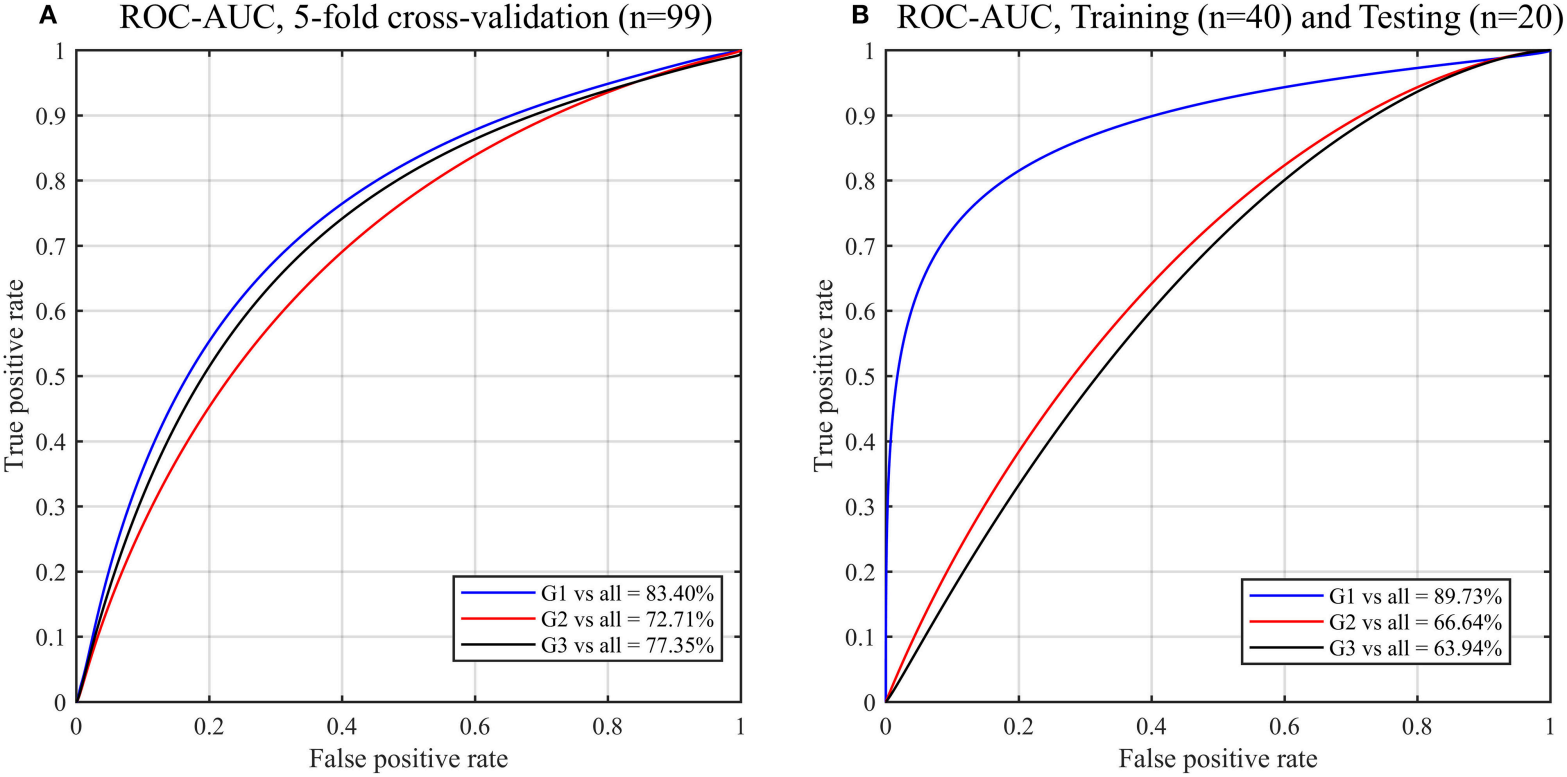
ROC curves and AUC obtained by the random forest (RF) models for predicting Gleason score of PCa patients using the radiomic features. Gleason score groups: G1 (group 1), G2 (group 2), G3 (group 3). **(A)** 5-fold cross validation, **(B)** Validation of the trained (*n* = 40; balanced classes) RF model by testing new datasets (*n* = 20).

Using all the radiomic features (i.e., 41 features) as input for the RF classifier model to predict the GS groups of the 99 PCa patients, the classifier accuracy was 81.82% (75.00–84.00%) for G1 patients, 66.67% (57.89–72.13%) for G2 patients and 74.75% (80.56–59.26%) for G3 patients (Supplementary Table 4). There was a higher value of the AUC of 83.40% to predict G1 compared to 72.71% and 77.35% when using radiomic features of G2 and G3, respectively (Figure 3A). The confusion matrix which reveals the RF classifier misclassification rate is shown in Supplementary Table 5. Correct classification of GS groups was achieved for 81/99 (18 G1 and 63 G2-G3), 66/99 (22 G2 and 44 G1-G3) and 74/99 (16 G3 and 58 G1-G2) of patients.

To validate our predictive model, we randomly assigned the 99 PCa patients to two datasets groups of balanced classes that were used as training (*n* = 40) and test (*n* = 20) datasets. Using the trained RF model to test the new datasets (*n* = 20), a higher value of the AUC of 89.73% to predict G1 compared to 66.64 and 63.94% when using radiomic features of G2 and G3, respectively (Figure 3B).

### Importance Features for Each of GS Groups

**Figure 4:**
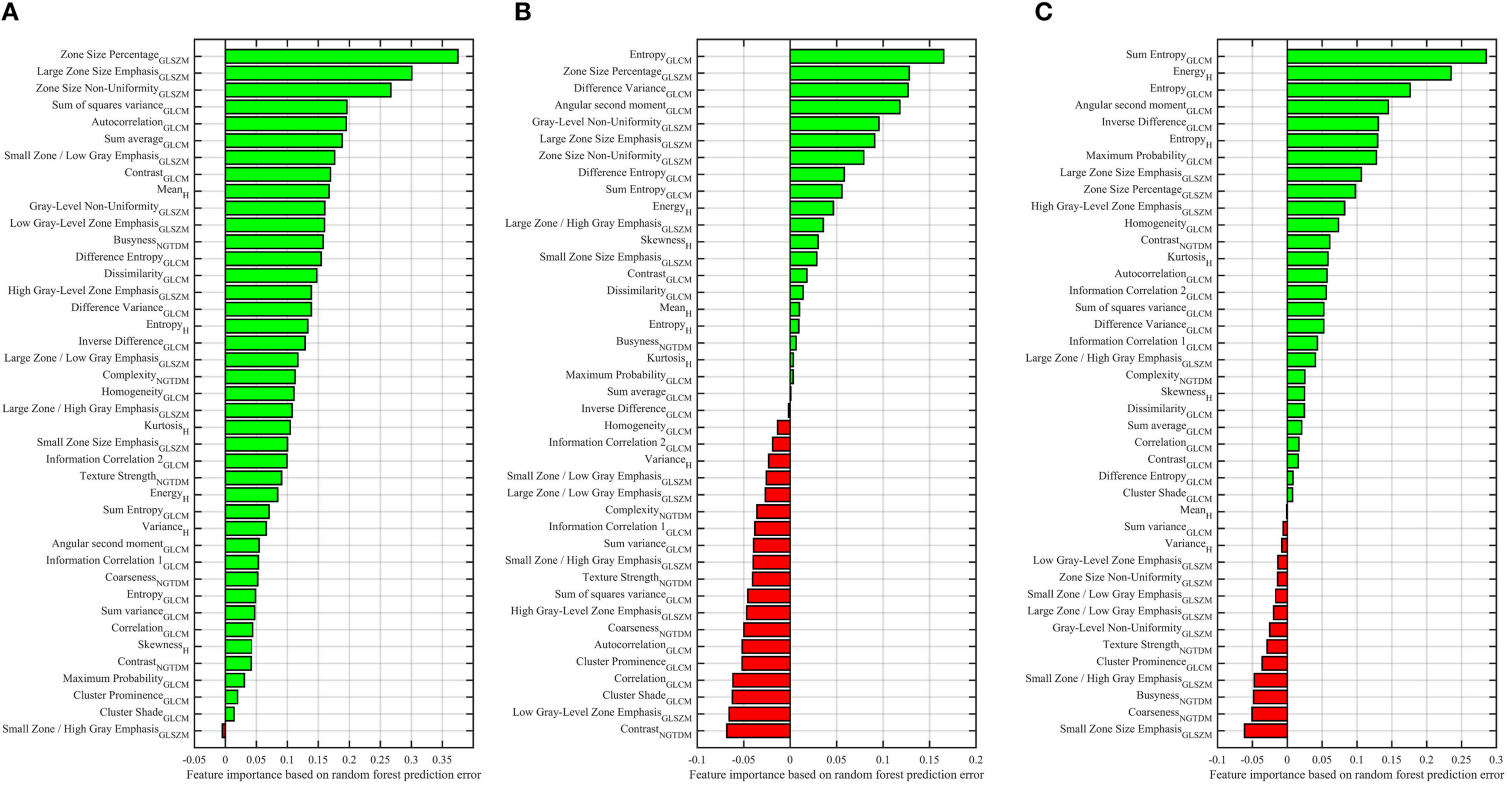
Importance of features for predicting the Gleason score of PCa patients, G1 vs. G2-G3 **(A)**, G2 vs. G1-G3 **(B)**, G3 vs. G1-G2 **(C)**. Reported values correspond to the average increase in prediction error obtained by permuting the values of individual features across out-of-bag observations (46). Green and red bars represent the positive and negative impact for predicting the Gleason score groups.

Features based on the RF classifier model for classifying patients into GS groups are depicted in Figure 4. We found that 40/41 radiomic features have an importance value >0, green bars, to classify G1 from G2 and G3 patients (Figure 4A). The most important features contributing to G1 identification were zone size percentage, large zone size emphasis, and zone size non-uniformity.

To distinguish G2 from G1 and G3, Figure 4B, 21 radiomic features had an importance value >0. We noticed that the entropy was the most dominant feature for predicting the G2 group. While 20 features had a negative importance value < 0, red bars. To distinguish G3 from G2 and G1, 27 features had an importance value >0 and the sum-entropy with energy-histogram were the most important features, while 14 features had a negative importance value, Figure 4C. The entropy and sum entropy features were the most important features to predict the G2 and G3, respectively. These features (i.e., entropy and sum entropy) describe tissue heterogeneity and measure the randomness of texture within the PCa region.

### Features Analysis for Predicting GS

**Figure 5:**
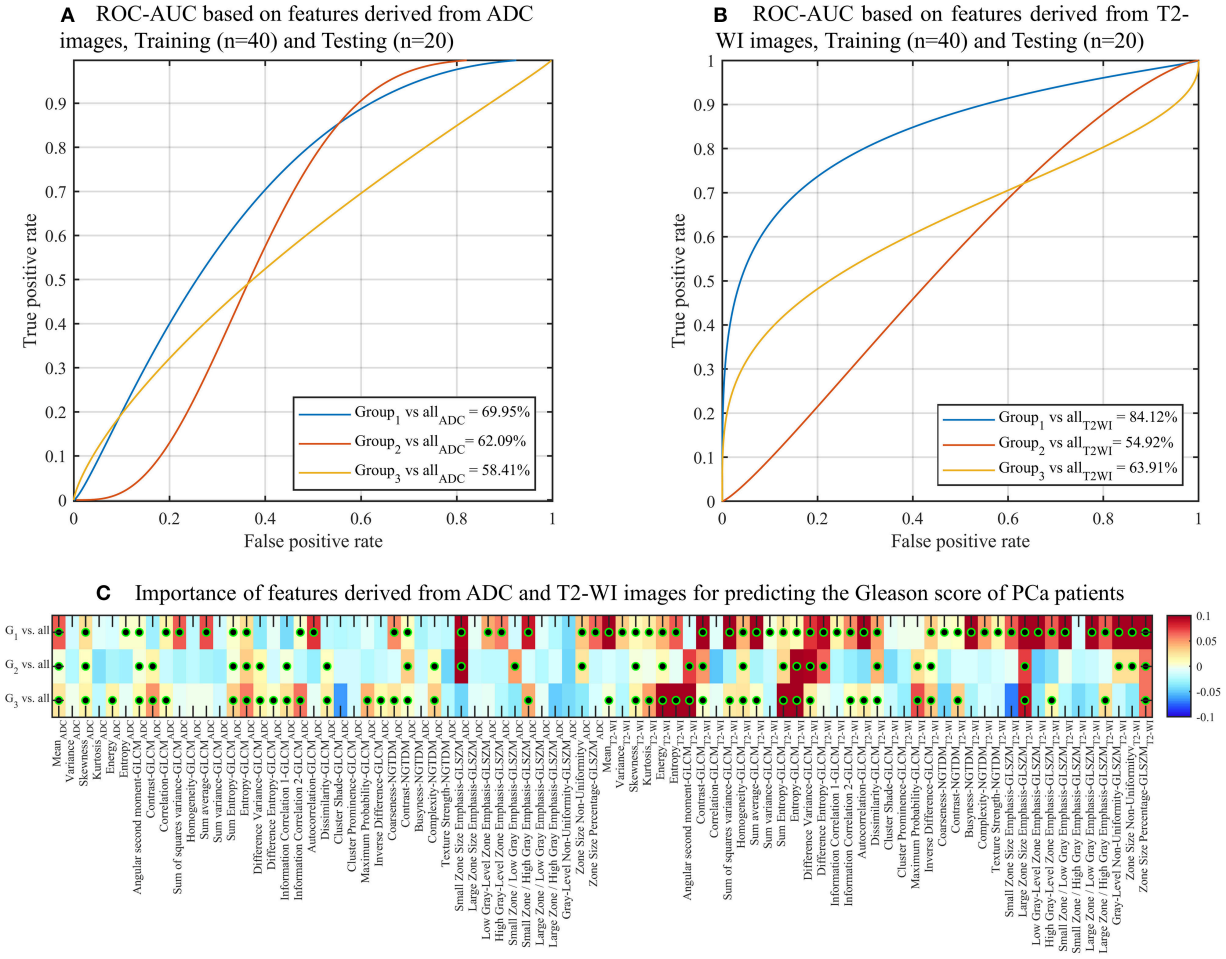
ROC curves and AUC obtained by RF models for predicting Gleason Score of PCa patients using the features derived from ADC **(A)** and T2-WI **(B)** images. **(C)** Heatmap of importance value for features predicting the three Gleason score groups of PCa patients. Color-coded from minimum (dark blue) to maximum (dark red). Features with statistically significant correlation (i.e., corrected *p* < 0.05) are indicated with a black-green circle. Features with predictive value (importance >0) are indicated with a black-green circle.

To analyse the impact of the features in predicting the GS of patients, we repeated the classification (training/testing = 40/20) between GS groups using the features derived from ADC and T2-WI images. We found that the AUC values of GS < 6 and GS ≥ 7 using the features derived from T2-WI images are higher with 84.12 and 63.91% to those derived from ADC with 69.95 and 58.41%, respectively. While the AUC value of GS = 7 (or 3 + 4) using the features derived from ADC images is higher with 62.09% comparing to 54.92% that derived from T2-WI images (Figures 5A,B). In general, the most important features for predicting the GS are derived from T2-WI images (Figure 5C).

## Discussion

Clinicians are trained for the ability to diagnose malignant disease through the visual study of MRI scans. However, visual methods are subjective, prone to errors and low throughput, a challenge which is becoming more of a limitation as the burden on healthcare resources expand with the aging population. Radiomic analysis, involving feature extraction from many images with classifier techniques, can automatically predict the grade of cancer with a precision and speed beyond the scope of human visual analysis. Several studies have used radiomic features, derived from MR images, for computer-assisted diagnosis (29, 31, 32, 49, 50). In addition to providing basic diagnostic information, such analysis may also reveal insights into the underlying heterogeneity of cancers, making further investigation into the radiomic assessment of PCa a priority. Radiomics has the additional benefit of automation, which can reduce human effort and cost whilst preventing patient morbidity and mortality associated with misdiagnosis and under/over treatment. However, radiomic features most helpful in predicting GS of PCa to estimate the aggressivity of a tumor remain largely unexplored.

In this study, we used three different methods: (i) Kruskal-Wallis significance test, (ii) Spearman rank correlation coefficient, and (iii) RF classifier model to test whether radiomics can successfully identify GS of PCa patients. Comparison of radiomic features between the three groups of GS revealed three radiomic features (i.e., large zone size emphasis, zone size non-uniformity and zone size percentage) with the capacity to discriminate between GS groups with a corrected *p* < 0.05. The similar three features were moderately correlated with GS groups with a corrected *p* < 0.05. Our findings confirm that the three features were associated with GS groups of PCa.

Binary classification using RF model demonstrated that similar features which were previously shown to be significantly correlated with GS groups are the most important (i.e., dominant) features for the prediction of patients with a GS = 6. These three features describe the homogeneity of the images through the size of uniform voxel regions in different PCa lesions. However, entropy and sum entropy features were demonstrated to have the greatest importance for predicting G2 (GS = [3 + 4]) and G3 (GS = [4 + 3, 8, 9, 10]) of PCa patients, respectively. Specifically, entropy and sum entropy features describe the randomness of the texture or the abnormalities of the PCa regions. The highest values of entropy features in G2 and G3 are linked to the abnormality in texture (i.e., heterogeneity) that is related to tumor (i.e., PCa) aggressiveness.

Our findings are consistent with several previous studies that utilized texture analysis. Haralick's texture features were demonstrated to be useful for PCa detection and GS assessment. Specifically, GS was associated with higher entropy features (27). Combined analysis of T2-WI images and MRS images demonstrated the feasibility for radiomics to discriminate between benign vs. cancerous and high vs. low GS using 29 preoperative mpMRI (i.e., T2-WI and MRS) (18). We observed that several previous studies have focused on the classification between the structure of benign and cancerous regions (51). This is consistent with our study in considering the Gleason score as the baseline indicator for classifying non-cancerous prostate from malignant cancers.

Our study has several limitations. Our analysis was performed on a retrospective analysis of a small group of patients (*n* = 99), including ADC and T2-WI MRI images only. More image modalities, such as proton density-weighted (PD-W) and dynamic contrast enhanced (DCE) could potentially improve the performance metrics for predicting the GS. Then, the results require external validation on a larger scale prior to broader clinical application. We considered only 41 different image-based features including first- and second-order textures that derived from sub-volume ROI without considering the manual segmentation to eliminate any bias resulting from inter-reader variability. PIRADS is an extensively studied and validated system, and any potential replacement needs to be compared to it as the current imaging standard.

Future work could explore the shape features (e.g., volume) of the full PCa area. Machine learning techniques, such as deep radiomics (52) based on convolutional neural networks could be also employed to learn discriminative features in a more data-driven manner.

## Conclusions

In this study, we presented the radiomic features that were computed from both ADC and T2-WI images for the discrimination between three groups of GS and classifying these groups using the RF classifier model. Our results suggest that only three features (i.e., zone size percentage, large zone size emphasis, and zone size non-uniformity) are able to identify groups of GS and significantly correlate each group. These three features appeared to be the most important to predict GS ≤ 6, while the sum entropy was the most important feature to predict a GS ≥ 7 (4 + 3). Radiomic analysis has the potential to be used as a non-invasive test to predict GS for patients with PCa and therefore, further prospective studies are warranted to validate and confirm our findings.

## Author Contributions

AC performed the experiments, analyzed data and wrote the paper. All authors reviewed the paper and gave final approval of the manuscript.

### Conflict of Interest Statement

The authors declare that the research was conducted in the absence of any commercial or financial relationships that could be construed as a potential conflict of interest.
