# Supplementary material for: Predicting Gleason Score of Prostate Cancer Patients Using Radiomic Analysis
Source: Front Oncol. 2018 Dec 18;8:630. doi: 10.3389/fonc.2018.00630 (PMC6305278; doi:10.3389/fonc.2018.00630)
Supplement: Supplementary file 1 [file Data_Sheet_1.pdf]

Supplementary Material

Predicting Gleason Score of Prostate Cancer Patients using Radiomic Analysis

Ahmad Chaddad[1,2*], Tamim Niazi[1*], Stephan Probst[3], Franck Bladou[4], Maurice Anidjar[4], Boris Bahoric[1]

[1]Division of Radiation Oncology, McGill University, Montreal, Quebec, Canada

[2]Department of automated production engineering, ETS, Montreal, Canada

[3]Division of Nuclear Medicine, McGill University, Montreal, Canada

[4]Depatment of Urology, McGill University, Montreal, Canada

*** Correspondence authors:**
Ahmad Chaddad & Tamim Niazi
Department of radiation oncology,
McGill University Health Center,
1001 Boulevard Décarie,
Montréal, Québec, H4A 3J1, Canada
Tel : +1-514-619-0751
Email: ahmad.chaddad@mail.mcgill.ca or tniazi@jgh.mcgill.ca

1 Supplementary Tables

Supplementary Table 1: PCa patients (n=99 cases)

ProstateX-0000	ProstateX-0026	ProstateX-0055	ProstateX-0094	ProstateX-0110	ProstateX-0123	ProstateX-0139	ProstateX-0164	ProstateX-0183	ProstateX-0195
ProstateX-0001	ProstateX-0028	ProstateX-0067	ProstateX-0095	ProstateX-0111	ProstateX-0124	ProstateX-0141	ProstateX-0165	ProstateX-0184	ProstateX-0196
ProstateX-0002	ProstateX-0031	ProstateX-0069	ProstateX-0097	ProstateX-0114	ProstateX-0125	ProstateX-0142	ProstateX-0168	ProstateX-0186	ProstateX-0197
ProstateX-0004	ProstateX-0035	ProstateX-0071	ProstateX-0099	ProstateX-0115	ProstateX-0126	ProstateX-0143	ProstateX-0169	ProstateX-0187	ProstateX-0198
ProstateX-0005	ProstateX-0037	ProstateX-0076	ProstateX-0101	ProstateX-0116	ProstateX-0127	ProstateX-0145	ProstateX-0172	ProstateX-0188	ProstateX-0199
ProstateX-0014	ProstateX-0040	ProstateX-0077	ProstateX-0102	ProstateX-0117	ProstateX-0128	ProstateX-0148	ProstateX-0173	ProstateX-0190	ProstateX-0200
ProstateX-0015	ProstateX-0041	ProstateX-0078	ProstateX-0103	ProstateX-0118	ProstateX-0129	ProstateX-0150	ProstateX-0174	ProstateX-0191	ProstateX-0201
ProstateX-0016	ProstateX-0046	ProstateX-0082	ProstateX-0104	ProstateX-0119	ProstateX-0131	ProstateX-0152	ProstateX-0179	ProstateX-0192	ProstateX-0202
ProstateX-0018	ProstateX-0053	ProstateX-0084	ProstateX-0105	ProstateX-0121	ProstateX-0133	ProstateX-0153	ProstateX-0181	ProstateX-0193	ProstateX-0203
ProstateX-0019	ProstateX-0054	ProstateX-0092	ProstateX-0106	ProstateX-0122	ProstateX-0136	ProstateX-0162	ProstateX-0182	ProstateX-0194	

Supplementary Table 2: Patients characteristics and Gleason score

<table>
<tr><th>Gleason (n)</th><th colspan="2">Gleason score</th><th>PCa zone</th><th>This study</th></tr>
<tr><td>Grade Group 1 (30)</td><td colspan="2">≤ 6</td><td>PZ=10; AS=14; TZ=6</td><td>G1 (30)</td></tr>
<tr><td>Grade Group 2 (39)</td><td colspan="2">3+4 = 7</td><td>PZ=19; AS=15; TZ=5</td><td>G2 (39)</td></tr>
<tr><td>Grade Group 3 (17)</td><td rowspan="3">≥ 7</td><td>4+3=7 or</td><td>PZ=6; AS=8; TZ=3</td><td rowspan="3">G3 (30)</td></tr>
<tr><td>Grade Group 4 (7)</td><td>4+4=8; 3+5=8;5+3=8</td><td>PZ=3; AS=3; TZ=1</td></tr>
<tr><td>Grade Group 5 (6)</td><td>9 or 10</td><td>PZ=2; AS=4; TZ=0</td></tr>
</table>

Supplementary Table 3: Description of features computed within the sub-volume of PCa tumor

Features	Description
**Histogram (first order features): These features are derived from the first order statistics and provide information related to the gray-level distribution of the ROIs**	
**Average (Mean)**	The average gray level intensity
**Variance**	Measures the spread distribution about the average
**Skewness**	Measures the asymmetry of the distribution of values about the average value
**Kurtosis**	Measures the peakedness of the distribution of values in the image
**Energy**	Measures the magnitude of voxel values
**Entropy**	Describes the randomness in the image values
**Grey-level co-occurrence matrix (GLCM): These texture features use second order statistics to characterize the spatial relationship between intensity values within** ROIs	
**Angular second moment**	Measures the textural uniformity that is pixel pair repetitions. It detects disorders in textures
**Contrast**	Measures the local intensity variation
**Correlation**	Describes the linear dependency of gray level values to their respective voxels in the GLCM
**Sum of squares variance**	Measures the distribution of neighboring intensity level pairs about the average of intensity level in the GLCM
**Homogeneity**	Measures the closeness of the distribution of elements in the GLCM to the GLCM diagonal
**Sum-average**	Measures the relationship between occurrences of pairs with lower intensity values and occurrences of pairs with higher intensity values
**Sum-variance**	Describes the weights elements that differ from the average value of the GLCM.
**Sum-entropy**	Represents the sum of neighborhood intensity value differences.
**Entropy**	Describes the randomness in the GLCM
**Difference variance**	Measure of heterogeneity that places higher weights on differing intensity level pairs that deviate more from the mean.
**Difference entropy**	Measure of the randomness/variability in neighborhood intensity value differences.
**Information measure of correlation 1**	Measures the differences of randomness (entropy)
**Information measure of correlation 2**	Measure the differences of randomness using exponential formula
**Autocorrelation**	measure of the magnitude of the fineness and coarseness of texture
**Dissimilarity**	Describes the contrast of local region
**Cluster shade**	Measure of the skewness and uniformity of the GLCM
**Cluster prominence**	Measure of the skewness and asymmetry of the GLCM
**Maximum probability**	Represents the occurrences of the most predominant pair of neighboring intensity values.
**Inverse difference**	Measures of the local homogeneity of an image
**Neighborhood grey-tone difference matrix (NGTDM): These features use higher-order statistics to measure intensity differences between neighbor voxels**	
**Coarseness**	Describes the texture uniformity
**Contrast**	Describes the spatial intensity change and the overall gray level dynamic range.
**Busyness**	Measures the change from a pixel/voxel to its neighbour.
**Complexity**	An image is considered complex when it has several rapid changes in gray level intensity.
**Texture Strength**	An image is considered strength when it has a slow change in intensity but more large coarse differences in gray level intensities.
**Grey-level zone size matrix (GLSZM/ GLZM): These texture features characterize the size of uniform voxel regions, called zones.**	
**Small zone size emphasis**	Describes the fine textures.
**Large zone size emphasis**	Describes the coarse textures.
**Low gray-level zone emphasis**	Measures the distribution of lower gray-level size zones, with a higher value indicating a greater proportion of lower gray-level values and size zones in the image
**High gray-level zone emphasis**	Measures the distribution of the higher gray-level values, with a higher value indicating a greater proportion of higher gray-level values and size zones in the image.
**Small zone / low gray emphasis**	Describes the joint distribution of smaller size zones with lower gray-level values.
**Small zone / high gray emphasis**	Describes the joint distribution of smaller size zones with higher gray-level values.
**Large zone / low gray emphasis**	Describes the joint distribution of larger size zones with lower gray-level values.
**Large zone / high gray emphasis**	Describes the joint distribution of larger size zones with higher gray-level values.
**Gray level non-uniformity**	Measures the variability of gray-level intensity values in the image, with a lower value indicating more homogeneity in intensity values.
**Zone Size Non-Uniformity**	Measures the variability of size zone volumes in the image, with a lower value indicating more homogeneity in size zone volumes.
**Zone Size Percentage**	Measures the coarseness of the texture by taking the ratio of number of zones and number of voxels in the ROI

Supplementary Table 4: Performance metrics (%) for predicting the groups of Gleason score

Metrics	G1 vs. all	G2 vs. all	G3 vs. all
**Classifier accuracy**	81.82	66.67	74.75
**NPV**	75.00	57.89	80.56
**PPV**	84.00	72.13	59.26
**AUC**	83.40	72.71	77.35

Supplementary Table 5: The confusion matrix for a binary classification

	G1 (n=30)	G2-G3 (n=69)		G2 (n=39)	G1-G3 (n=60)		G3 (n=30)	G1-G2 (n=69)
**G1**	18	12	**G2**	22	17	**G3**	16	14
**G2-G3**	6	63	**G1-G3**	16	44	**G1-G2**	11	58

2 Supplementary Figures

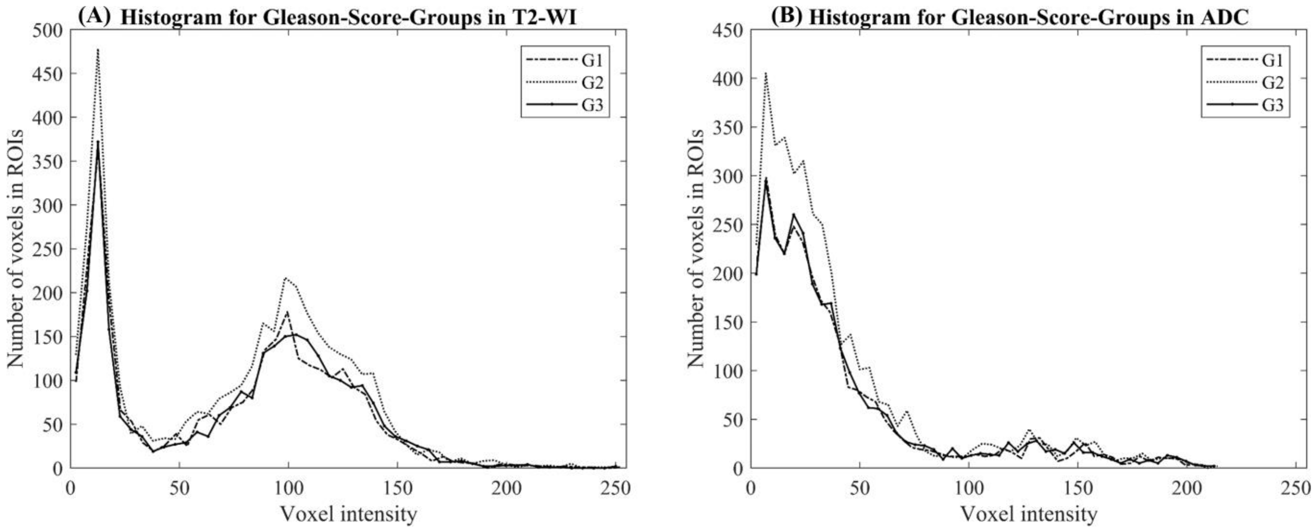

Supplementary Figure 1. Histogram of ROIs derived from the Gleason Score Groups (i.e. G1, G2 and G3) of PCa patients. (A) T2-WI and (B) ADC.

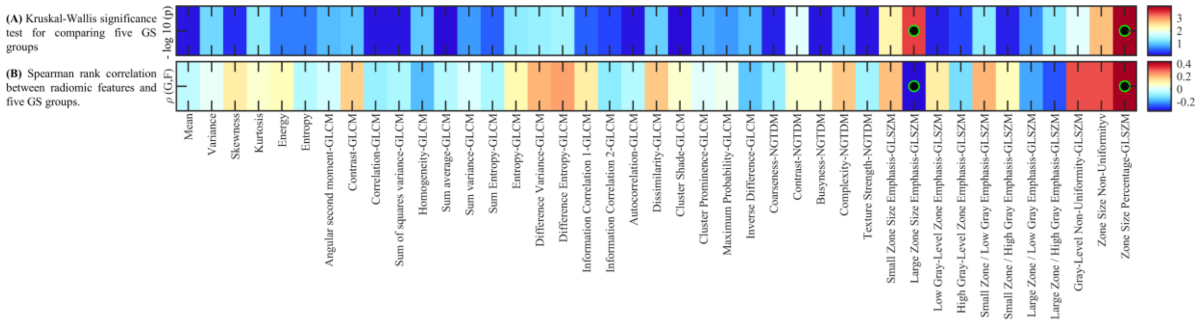

Supplementary Figure 2. (A) Heatmap of the Kruskal-Wallis significance test p values (-$\log_{10}$ scale) using radiomic features to identify patients of five groups of Gleason scores. (B) Spearman rank correlation between features value and five groups of Gleason score, color-coded from minimum (dark blue) to maximum (dark red). In (A) and (B), significant features are labeled with a black-green circle (corrected p <0.05).
